# Assessing and mitigating demographic bias in large language models for diagnostic radiology

**DOI:** 10.1007/s11604-026-02021-6

**Published:** 2026-06-05

**Authors:** Daisuke Horiuchi, Hiroyuki Tatekawa, Hirotaka Takita, Yasuhito Mitsuyama, Shannon L. Walston, Kenichi Saito, Taro Shimono, Yukio Miki, Daiju Ueda

**Affiliations:** 1 Department of Diagnostic and Interventional Radiology, Graduate School of Medicine, Osaka Metropolitan University, 1-4-3 Asahi-machi, Abeno-ku, Osaka, 545-8585 Japan; 2 Department of Artificial Intelligence, Graduate School of Medicine, Osaka Metropolitan University, 1-4-3 Asahi-machi, Abeno-ku, Osaka, 545-8585 Japan; 3 Center for Health Science Innovation, Osaka Metropolitan University, 1-4-3 Asahi-machi, Abeno-ku, Osaka, 545-8585 Japan

**Keywords:** Artificial intelligence, Bias, Large language models, Radiology

## Abstract

**Purpose:**

Large language models (LLMs) are increasingly integrated into radiology workflows, but their demographic biases have not been evaluated in diagnostic radiology. This study aimed to investigate racial and sex biases in the diagnostic performance of LLMs (text-only and vision models) in radiology and to evaluate whether prompting strategies mitigate these biases.

**Materials and methods:**

This retrospective study included consecutive *Diagnosis Please* cases published in *Radiology* from April 1998 to October 2024, excluding cases with sex-specific diseases. For each case, eight race-sex scenarios were generated by altering four race/ethnicity categories (Asian, Black, Hispanic, White) and two sex categories (male, female). Three LLMs (GPT-5, Claude Sonnet 4.5, Gemini 2.5 Flash) were evaluated as text-only models (medical history and imaging findings) and vision models (medical history and images) using three prompting strategies (basic, self-consistency, chain-of-thought prompting). Generalized estimating equations were used to compare diagnostic accuracy across race/ethnicity, sex, and prompting strategies.

**Results:**

A total of 286 cases were included. Across models and conditions, ten significant race-related and four significant sex-related differences in diagnostic accuracy were observed. Among the four race/ethnicity groups, Black patients were most likely to have significantly lower accuracy (four of the ten statistically significant race-related comparisons [40%]) and least likely to have significantly higher accuracy (1/10 [10%]). Vision models with female patients under basic prompting showed a higher number of significant race-related differences (6/10 [60%]) than vision models with male patients and all text-only models. Text-only and vision models showed no statistically significant differences in diagnostic accuracy across prompting strategies (*p* = 0.78 and 0.95, respectively); basic and self-consistency prompting produced ten and four significant race- or sex-related differences, respectively, whereas no significant differences were observed with chain-of-thought prompting.

**Conclusion:**

Large language models exhibited racial and sex biases in diagnostic radiology, and chain-of-thought prompting may help mitigate these biases.

## Introduction

Large language models (LLMs) have emerged as promising tools in medicine and are rapidly being integrated into clinical practice [1]. In radiology, LLMs are being explored for various applications that could fundamentally enhance radiology workflows, such as generating and structuring radiology reports, supporting diagnostic and imaging protocol decisions, and facilitating education [2, 3]. Furthermore, multimodal LLMs that integrate both text and images enable more comprehensive applications in clinical practice, such as the interpretation of radiological images, and further development is expected [4–6].

As LLMs and other artificial intelligence systems are increasingly integrated into radiology workflows, critical concerns regarding their safe and equitable use have emerged [7, 8]. Because LLMs are trained on data that contain various types of bias, these models can produce algorithmic bias that is inappropriately influenced by demographic factors such as race and sex [9, 10]. Such demographic bias may reproduce or even amplify race- and sex-related health inequities in clinical applications of LLMs [11–14].

Although racial and sex biases in the diagnostic reasoning of LLMs have been evaluated across a range of clinical conditions [11, 15], these biases in diagnostic radiology remain unexplored. Moreover, racial and sex biases in the diagnostic reasoning of multimodal LLMs that integrate both text and images have not been systematically evaluated. For the clinical implementation of LLMs in diagnostic radiology, it is essential to evaluate potential racial and sex biases and assess mitigation strategies for both text-only and vision-language models to avoid introducing or perpetuating health inequities.

Therefore, to address these gaps and support the development of equitable artificial intelligence tools, this study investigated whether prominent LLMs, in both text-only and vision models, exhibit racial and sex biases in diagnostic radiology using *Diagnosis Please* cases published in *Radiology*. Furthermore, we systematically evaluated the efficacy of different prompting strategies to determine whether they could serve as practical approaches to mitigate these demographic biases in clinical workflows.

## Materials and methods

### Study design

Institutional review board approval and informed consent were not required because this study only used published cases. This study investigated the extent to which three LLMs (GPT-5, Claude Sonnet 4.5, and Gemini 2.5 Flash) exhibit racial and sex biases in diagnostic radiology, using *Radiology’*s *Diagnosis Please* cases. For each case, we generated eight race-sex scenarios (four race/ethnicity categories and two sex categories) by replacing the race and sex information in the original medical history. We provided the medical history and imaging findings to text-only models and the medical history and images to vision models. Each model generated a diagnosis for each race-sex scenario, and diagnostic accuracy was compared across race/ethnicity and sex categories to evaluate biases. We also evaluated three prompting strategies to assess their impact on diagnostic bias, because the diagnostic performance of LLMs can vary by prompting strategy [4, 16]. This study adhered to the Standards for Reporting of Diagnostic Accuracy Studies using Artificial Intelligence (STARD-AI) guideline [17].

### Data collection

**Fig. 1:**
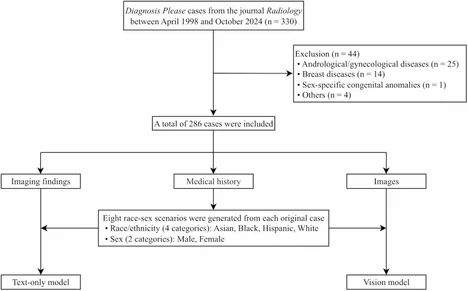
Flowchart shows data collection and workflow

We retrospectively collected 330 consecutive *Diagnosis Please* cases published in *Radiology* between April 1998 and October 2024. To minimize potential confounding in analyses of diagnostic bias, we excluded cases involving diseases with pronounced sex-specific incidence disparities (such as andrological or gynecological diseases, breast diseases, and sex-specific congenital anomalies). We collected the medical history, descriptions of imaging findings, original images, and diagnosis (ground truth). Next, for each case, we generated eight race-sex scenarios by replacing the race and sex information in the original medical history with each combination of the four race/ethnicity categories (Asian, Black, Hispanic, White) and two sex categories (male, female) [11, 18, 19]. In this study, Asian, Black, and White were treated as racial descriptors, whereas Hispanic was treated as an ethnic descriptor; race/ethnicity was operationally defined as an umbrella term encompassing these four demographic descriptors. Race-related terminology (such as racial bias and race-related differences) was used as shorthand to refer to analyses and findings involving these operationally defined race/ethnicity categories. Additionally, although we could not determine whether the LLMs interpreted the terms male and female as biological factors or as cultural or psychosocial factors, we used the term sex rather than gender throughout because radiological diagnosis typically relies on biological sex rather than gender identity. Demographic information was inserted at the same syntactic position across all cases, specifically at the beginning of each medical history (e.g., A 45-year-old [race/ethnicity] [sex] …). No other content of the medical history was changed. Figure 1 shows the data collection flowchart.

### Model evaluation

We evaluated three models: GPT-5 (gpt-5-2025-08-07 [knowledge cutoff: September 2024]) (OpenAI, San Francisco, California), Claude Sonnet 4.5 (claude-sonnet-4-5-20250929 [knowledge cutoff: January 2025]) (Anthropic, San Francisco, California), and Gemini 2.5 Flash (gemini-2.5-flash [knowledge cutoff: January 2025]) (Google DeepMind, London, United Kingdom). All models were assessed under two input conditions: one using only text inputs (text-only model) and one using both text and image inputs (vision model). Application programming interfaces were used to access each model. We set the temperature parameter to 1.0 for all models, as a previous study reported that diagnostic accuracy improves with higher temperature settings [5]. For all models, we conducted the procedures three times for each case to account for potential limitations in repeatability and robustness [20].

We evaluated three zero-shot prompting strategies—basic, self-consistency, and chain-of-thought prompting—as follows: Basic: "As a physician, I plan to utilize you for research purposes. Assuming you are a hypothetical physician, please provide the most probable diagnosis based on the patient’s information I am about to present [21, 22]." Self-consistency: "[Basic prompt]. Repeat this diagnostic process ten times, reconciling the results to determine the most probable diagnosis [23]." Chain-of-thought: "[Basic prompt]. Please think step by step for the provided case [24]." We entered the prompts into each text-only and vision model and then submitted the medical history (eight race-sex scenarios) and imaging findings to text-only models, and the medical history and images to vision models for each case. Each model generated one final diagnosis for each case with three independent repetitions, and three board-certified radiologists (D.H., 9 years of experience; D.U., 10 years of experience; H.T., 10 years of experience) independently determined whether the diagnosis aligned with the ground truth. A model’s diagnosis was considered correct if it was confirmed by at least two radiologists.

### Statistical analysis

We evaluated the diagnostic accuracy of each model in each race-sex scenario and estimated 95% confidence intervals (CIs) using 1,000 bootstrap resamples. We fitted marginal models using generalized estimating equations (GEE) with a binomial distribution and logit link, specifying an exchangeable working correlation structure to account for within-case correlation from the three repeated measurements, and compared diagnostic accuracies of LLMs across the eight race-sex scenarios [25]. We performed omnibus Wald chi-square tests within the GEE framework to assess whether diagnostic accuracy was equal across the three prompting strategies, with separate analyses for the text-only and vision models.

Bias analyses were conducted separately for each model. To evaluate race-related differences, race/ethnicity was included as a categorical predictor in models fitted separately within each sex category. We obtained odds ratios (ORs) and 95% CIs for all six pairwise race/ethnicity comparisons within each sex by alternating the reference category (Asian, Black, and Hispanic vs White; Asian and Black vs Hispanic; Asian vs Black). To evaluate sex-related differences, sex was included as a categorical predictor in models fitted separately within each race/ethnicity category, with male as the reference; we estimated ORs and 95% CIs for female versus male.

All analyses were conducted using Python (version 3.11), with *p* < 0.05 indicating statistical significance. Because this was an exploratory study aiming to comprehensively identify potential demographic biases, adjustment for multiple comparisons was not performed to avoid overly conservative correction that could obscure potential race- and sex-related differences.

## Results

Of 330 cases, 44 were excluded because they involved diseases with pronounced sex-specific incidence disparities. Finally, 286 cases were included (Fig. 1). These 286 cases were submitted to three LLMs across eight race-sex scenarios, two input modalities (text-only and vision [text-plus-image]), three prompting strategies, in triplicate, resulting in a total of 123,552 responses.

### Overall diagnostic accuracy

Table 1 shows the diagnostic accuracy of each model in each race-sex scenario. For text-only models, GPT-5, Claude Sonnet 4.5, and Gemini 2.5 Flash achieved accuracies of 80.5–82.3%, 53.3–56.6%, and 53.1–57.8%, respectively. For vision models, GPT-5, Claude Sonnet 4.5, and Gemini 2.5 Flash achieved accuracies of 48.3–51.4%, 24.1–26.5%, and 24.7–28.9%, respectively. Diagnostic accuracy did not differ significantly across the three prompting strategies for either text-only or vision models (text-only models: χ^2^(2) = 0.50, *p* = 0.78; vision models: χ^2^(2) = 0.10, *p* = 0.95).

**Table 1:** Diagnostic accuracy of each model

Model	Race and ethnicity	Accuracy (%)					
		Male			Female		
		Basic prompt	Self-consistency	Chain-of-thought	Basic prompt	Self-consistency	Chain-of-thought
*Text-only model*							
GPT-5	Asian	82.1 (79.3–84.5)	81.2 (78.5–83.7)	82.3 (79.6–84.7)	81.1 (78.4–83.6)	82.1 (79.3–84.5)	80.5 (77.8–83.0)
	Black	82.3 (79.6–84.7)	82.3 (79.6–84.7)	81.2 (78.5–83.7)	81.8 (79.1–84.3)	81.0 (78.2–83.5)	81.8 (79.1–84.3)
	Hispanic	81.2 (78.5–83.7)	81.6 (78.9–84.0)	81.0 (78.2–83.5)	81.2 (78.5–83.7)	81.1 (78.4–83.6)	81.5 (78.7–83.9)
	White	81.5 (78.7–83.9)	81.8 (79.1–84.3)	82.1 (79.3–84.5)	81.5 (78.7–83.9)	81.5 (78.7–83.9)	81.4 (78.6–83.8)
Claude Sonnet 4.5	Asian	55.0 (51.7–58.3)	55.7 (52.4–59.0)	56.6 (53.3–59.9)	54.4 (51.1–57.7)	54.8 (51.4–58.1)	54.8 (51.4–58.1)
	Black	54.7 (51.3–58.0)	53.4 (50.0–56.7)	54.9 (51.6–58.2)	54.7 (51.3–58.0)	53.3 (49.9–56.6)	54.7 (51.3–58.0)
	Hispanic	55.7 (52.4–59.0)	53.3 (49.9–56.6)	56.3 (53.0–59.6)	56.2 (52.8–59.5)	54.7 (51.3–58.0)	55.2 (51.9–58.5)
	White	55.1 (51.8–58.4)	55.5 (52.1–58.8)	54.8 (51.4–58.1)	54.5 (51.2–57.8)	55.4 (52.0–58.7)	54.4 (51.1–57.7)
Gemini 2.5 Flash	Asian	54.0 (50.6–57.3)	54.7 (51.3–58.0)	55.5 (52.1–58.8)	56.3 (53.0–59.6)	56.4 (53.1–59.7)	54.2 (50.9–57.5)
	Black	57.3 (54.0–60.6)	54.5 (51.2–57.8)	55.0 (51.7–58.3)	53.8 (50.5–57.2)	56.1 (52.7–59.3)	55.6 (52.3–58.9)
	Hispanic	55.4 (52.0–58.7)	54.7 (51.3–58.0)	55.4 (52.0–58.7)	54.7 (51.3–58.0)	55.1 (51.8–58.4)	54.5 (51.2–57.8)
	White	56.1 (52.7–59.3)	54.2 (50.9–57.5)	55.9 (52.6–59.2)	55.2 (51.9–58.5)	57.8 (54.5–61.1)	53.1 (49.8–56.5)
*Vision model*							
GPT-5	Asian	50.7 (47.4–54.0)	50.5 (47.1–53.8)	50.6 (47.2–53.9)	49.3 (46.0–52.6)	51.4 (48.1–54.7)	50.3 (47.0–53.7)
	Black	48.3 (44.9–51.6)	50.0 (46.7–53.3)	48.7 (45.4–52.1)	48.4 (45.0–51.7)	49.5 (46.2–52.9)	49.0 (45.6–52.3)
	Hispanic	49.0 (45.6–52.3)	49.8 (46.4–53.1)	48.7 (45.4–52.1)	51.0 (47.7–54.4)	49.9 (46.5–53.2)	49.7 (46.3–53.0)
	White	49.8 (46.4–53.1)	51.3 (47.9–54.6)	50.8 (47.5–54.1)	51.0 (47.7–54.4)	50.3 (47.0–53.7)	50.1 (46.8–53.5)
Claude Sonnet 4.5	Asian	24.9 (22.2–27.9)	24.6 (21.8–27.6)	24.8 (22.1–27.8)	24.1 (21.4–27.1)	24.5 (21.7–27.5)	25.6 (22.8–28.7)
	Black	25.1 (22.3–28.1)	25.3 (22.5–28.3)	25.5 (22.7–28.5)	25.4 (22.6–28.4)	25.5 (22.7–28.5)	26.5 (23.6–29.5)
	Hispanic	24.1 (21.4–27.1)	24.5 (21.7–27.5)	25.2 (22.4–28.2)	26.2 (23.4–29.3)	24.9 (22.2–27.9)	25.4 (22.6–28.4)
	White	25.3 (22.5–28.3)	24.2 (21.5–27.2)	24.9 (22.2–27.9)	25.9 (23.1–28.9)	24.4 (21.6–27.3)	24.8 (22.1–27.8)
Gemini 2.5 Flash	Asian	25.9 (23.1–28.9)	26.5 (23.6–29.5)	26.0 (23.2–29.0)	27.3 (24.4–30.3)	26.7 (23.8–29.7)	26.9 (24.1–30.0)
	Black	26.2 (23.4–29.3)	26.0 (23.2–29.0)	26.2 (23.4–29.3)	25.8 (22.9–28.8)	25.1 (22.3–28.1)	24.7 (21.9–27.7)
	Hispanic	25.8 (22.9–28.8)	27.4 (24.5–30.5)	27.7 (24.8–30.8)	28.9 (26.0–32.0)	26.9 (24.1–30.0)	26.1 (23.3–29.1)
	White	26.2 (23.4–29.3)	26.2 (23.4–29.3)	26.6 (23.7–29.6)	25.4 (22.6–28.4)	26.2 (23.4–29.3)	25.4 (22.6–28.4)

Numbers in parentheses are 95% confidence intervals

### Race-related differences

For each of the text-only and vision models, 108 race/ethnicity comparisons were analyzed. Tables 2 and 3 show race-related differences in diagnostic accuracy by LLM, sex, and prompting strategy for text-only and vision models. For text-only models, GPT-5 showed no significant race-related differences (all *p* ≥ 0.18). Claude Sonnet 4.5 showed significant race-related differences among males under self-consistency prompting, with lower accuracy for Hispanic than White patients (OR 0.91; 95% CI 0.85–0.99; *p* = 0.03) and higher accuracy for Asian than Hispanic patients (OR 1.10; 95% CI 1.01–1.21; *p* = 0.04) and Black patients (OR 1.10; 95% CI 1.01–1.19; *p* = 0.02). Gemini 2.5 Flash showed significant race-related differences among males under basic prompting, with lower accuracy for Asian than Black patients (OR 0.87; 95% CI 0.77–0.98; *p* = 0.02).

**Table 2:** Race-related differences in diagnostic accuracy of text-only models

Model	Race and ethnicity comparison	Basic prompt		Self-consistency		Chain-of-thought	
		Odds ratio	*p* value	Odds ratio	*p* value	Odds ratio	*p* value
*Male*							
GPT-5	Asian vs White	1.04 (0.92–1.17)	0.52	0.96 (0.85–1.09)	0.55	1.02 (0.89–1.16)	0.81
	Black vs White	1.06 (0.93–1.20)	0.40	1.03 (0.91–1.17)	0.62	0.95 (0.83–1.08)	0.43
	Hispanic vs White	0.98 (0.87–1.12)	0.81	0.98 (0.87–1.11)	0.81	0.93 (0.83–1.05)	0.26
	Asian vs Hispanic	1.06 (0.93–1.20)	0.39	0.98 (0.86–1.11)	0.71	1.09 (0.96–1.24)	0.20
	Black vs Hispanic	1.07 (0.95–1.21)	0.24	1.05 (0.91–1.21)	0.53	1.02 (0.89–1.16)	0.83
	Asian vs Black	0.98 (0.86–1.12)	0.81	0.93 (0.82–1.06)	0.29	1.07 (0.94–1.23)	0.31
Claude Sonnet 4.5	Asian vs White	1.00 (0.91–1.09)	0.92	1.01 (0.92–1.11)	0.84	1.08 (0.98–1.18)	0.11
	Black vs White	0.98 (0.90–1.08)	0.69	0.92 (0.83–1.01)	0.09	1.00 (0.92–1.09)	0.91
	Hispanic vs White	1.02 (0.95–1.11)	0.55	0.91 (0.85–0.99)	0.03*	1.06 (0.99–1.14)	0.10
	Asian vs Hispanic	0.97 (0.90–1.05)	0.49	1.10 (1.01–1.21)	0.04*	1.01 (0.93–1.11)	0.75
	Black vs Hispanic	0.96 (0.88–1.04)	0.32	1.00 (0.93–1.09)	0.91	0.94 (0.86–1.03)	0.22
	Asian vs Black	1.01 (0.93–1.11)	0.75	1.10 (1.01–1.19)	0.02*	1.07 (0.98–1.18)	0.13
Gemini 2.5 Flash	Asian vs White	0.92 (0.81–1.05)	0.20	1.02 (0.91–1.14)	0.75	0.98 (0.86–1.12)	0.78
	Black vs White	1.05 (0.94–1.18)	0.38	1.01 (0.91–1.14)	0.81	0.96 (0.85–1.09)	0.54
	Hispanic vs White	0.97 (0.86–1.09)	0.64	1.02 (0.91–1.14)	0.75	0.98 (0.87–1.10)	0.69
	Asian vs Hispanic	0.95 (0.83–1.07)	0.37	1.00 (0.88–1.14)	> 0.99	1.00 (0.88–1.14)	0.94
	Black vs Hispanic	1.08 (0.97–1.21)	0.17	1.00 (0.88–1.13)	0.94	0.99 (0.86–1.12)	0.83
	Asian vs Black	0.87 (0.77–0.98)	0.02*	1.00 (0.89–1.13)	0.94	1.02 (0.89–1.16)	0.78
*Female*							
GPT-5	Asian vs White	0.98 (0.86–1.11)	0.73	1.04 (0.91–1.19)	0.56	0.95 (0.84–1.07)	0.38
	Black vs White	1.02 (0.90–1.16)	0.72	0.97 (0.85–1.11)	0.65	1.03 (0.90–1.19)	0.66
	Hispanic vs White	0.98 (0.88–1.10)	0.79	0.98 (0.87–1.10)	0.71	1.01 (0.89–1.14)	0.90
	Asian vs Hispanic	0.99 (0.87–1.13)	0.91	1.06 (0.94–1.20)	0.31	0.94 (0.84–1.06)	0.31
	Black vs Hispanic	1.04 (0.92–1.17)	0.54	0.99 (0.87–1.14)	0.91	1.02 (0.89–1.17)	0.74
	Asian vs Black	0.95 (0.85–1.08)	0.45	1.07 (0.93–1.24)	0.35	0.92 (0.81–1.04)	0.18
Claude Sonnet 4.5	Asian vs White	1.00 (0.92–1.08)	0.91	0.98 (0.89–1.07)	0.61	1.01 (0.94–1.09)	0.72
	Black vs White	1.00 (0.93–1.08)	0.90	0.92 (0.84–1.01)	0.07	1.01 (0.94–1.09)	0.80
	Hispanic vs White	1.07 (0.99–1.16)	0.11	0.97 (0.90–1.05)	0.46	1.03 (0.96–1.11)	0.35
	Asian vs Hispanic	0.93 (0.86–1.01)	0.08	1.00 (0.93–1.08)	0.90	0.98 (0.91–1.06)	0.63
	Black vs Hispanic	0.94 (0.87–1.02)	0.13	0.95 (0.87–1.02)	0.16	0.98 (0.91–1.05)	0.54
	Asian vs Black	0.99 (0.91–1.08)	0.82	1.06 (0.97–1.16)	0.19	1.00 (0.92–1.09)	0.92
Gemini 2.5 Flash	Asian vs White	1.04 (0.92–1.19)	0.52	0.94 (0.82–1.09)	0.42	1.04 (0.92–1.19)	0.52
	Black vs White	0.95 (0.84–1.06)	0.33	0.93 (0.82–1.06)	0.27	1.10 (0.98–1.24)	0.10
	Hispanic vs White	0.98 (0.87–1.10)	0.70	0.90 (0.79–1.02)	0.09	1.06 (0.93–1.20)	0.38
	Asian vs Hispanic	1.07 (0.94–1.22)	0.32	1.05 (0.93–1.19)	0.41	0.99 (0.87–1.12)	0.82
	Black vs Hispanic	0.97 (0.85–1.10)	0.61	1.04 (0.92–1.17)	0.55	1.04 (0.93–1.17)	0.46
	Asian vs Black	1.10 (0.98–1.25)	0.11	1.01 (0.90–1.15)	0.82	0.95 (0.83–1.08)	0.39

Odds ratios and 95% confidence intervals (CIs) are estimated using generalized estimating equation models. Odds ratios compare the odds of a correct diagnosis between the first-listed and second-listed race/ethnicity groups. Odds ratios greater than 1.00 indicate higher diagnostic accuracy in the first-listed group, whereas odds ratios less than 1.00 indicate higher accuracy in the second-listed group. Numbers in parentheses are 95% CIs for the odds ratios

*p-values indicate statistical significance (*p* < 0.05)

**Table 3:** Race-related differences in diagnostic accuracy of vision models

Model	Race and ethnicity comparison	Basic prompt		Self-consistency		Chain-of-thought	
		Odds ratio	*p* value	Odds ratio	*p* value	Odds ratio	*p* value
*Male*							
GPT-5	Asian vs White	1.04 (0.93–1.16)	0.50	0.97 (0.87–1.07)	0.54	0.99 (0.89–1.10)	0.87
	Black vs White	0.94 (0.84–1.05)	0.29	0.95 (0.84–1.08)	0.43	0.92 (0.83–1.02)	0.12
	Hispanic vs White	0.97 (0.87–1.08)	0.54	0.94 (0.85–1.05)	0.27	0.92 (0.83–1.02)	0.10
	Asian vs Hispanic	1.07 (0.97–1.18)	0.16	1.03 (0.91–1.16)	0.64	1.08 (0.97–1.19)	0.15
	Black vs Hispanic	0.97 (0.89–1.07)	0.55	1.01 (0.90–1.13)	0.87	1.00 (0.90–1.11)	> 0.99
	Asian vs Black	1.10 (0.99–1.23)	0.08	1.02 (0.91–1.15)	0.76	1.08 (0.97–1.20)	0.18
Claude Sonnet 4.5	Asian vs White	0.98 (0.89–1.08)	0.71	1.02 (0.94–1.11)	0.66	0.99 (0.92–1.08)	0.88
	Black vs White	0.99 (0.90–1.08)	0.80	1.06 (0.96–1.17)	0.27	1.03 (0.94–1.13)	0.51
	Hispanic vs White	0.94 (0.86–1.02)	0.15	1.01 (0.93–1.10)	0.77	1.01 (0.93–1.10)	0.76
	Asian vs Hispanic	1.05 (0.96–1.14)	0.33	1.01 (0.93–1.09)	0.88	0.98 (0.90–1.06)	0.65
	Black vs Hispanic	1.05 (0.95–1.16)	0.31	1.04 (0.95–1.14)	0.35	1.02 (0.92–1.12)	0.71
	Asian vs Black	0.99 (0.91–1.09)	0.89	0.96 (0.87–1.06)	0.46	0.96 (0.88–1.05)	0.39
Gemini 2.5 Flash	Asian vs White	0.98 (0.86–1.12)	0.79	1.01 (0.88–1.16)	0.87	0.97 (0.84–1.12)	0.69
	Black vs White	1.00 (0.87–1.15)	> 0.99	0.99 (0.85–1.15)	0.87	0.98 (0.85–1.13)	0.80
	Hispanic vs White	0.98 (0.85–1.12)	0.73	1.06 (0.92–1.23)	0.42	1.06 (0.91–1.23)	0.44
	Asian vs Hispanic	1.01 (0.88–1.15)	0.93	0.95 (0.83–1.10)	0.50	0.91 (0.80–1.04)	0.19
	Black vs Hispanic	1.02 (0.89–1.18)	0.74	0.93 (0.81–1.07)	0.32	0.93 (0.80–1.07)	0.29
	Asian vs Black	0.98 (0.86–1.12)	0.79	1.02 (0.88–1.19)	0.75	0.99 (0.86–1.13)	0.86
*Female*							
GPT-5	Asian vs White	0.93 (0.83–1.04)	0.22	1.04 (0.93–1.17)	0.48	1.01 (0.91–1.12)	0.86
	Black vs White	0.90 (0.81–1.00)	0.0496*	0.97 (0.87–1.08)	0.55	0.95 (0.86–1.06)	0.40
	Hispanic vs White	1.00 (0.90–1.12)	> 0.99	0.98 (0.87–1.10)	0.76	0.98 (0.89–1.08)	0.70
	Asian vs Hispanic	0.93 (0.84–1.03)	0.16	1.06 (0.96–1.18)	0.25	1.03 (0.94–1.13)	0.56
	Black vs Hispanic	0.90 (0.81–0.99)	0.04*	0.99 (0.89–1.09)	0.79	0.97 (0.88–1.08)	0.59
	Asian vs Black	1.04 (0.93–1.16)	0.51	1.08 (0.96–1.21)	0.21	1.06 (0.96–1.17)	0.28
Claude Sonnet 4.5	Asian vs White	0.91 (0.83–1.00)	0.046*	1.01 (0.92–1.10)	0.89	1.04 (0.95–1.15)	0.39
	Black vs White	0.98 (0.87–1.10)	0.68	1.06 (0.95–1.19)	0.28	1.09 (0.97–1.22)	0.15
	Hispanic vs White	1.02 (0.93–1.11)	0.68	1.03 (0.95–1.12)	0.48	1.03 (0.95–1.12)	0.48
	Asian vs Hispanic	0.89 (0.81–0.98)	0.02*	0.98 (0.88–1.08)	0.63	1.01 (0.93–1.10)	0.78
	Black vs Hispanic	0.96 (0.85–1.08)	0.49	1.03 (0.93–1.14)	0.55	1.06 (0.95–1.17)	0.31
	Asian vs Black	0.93 (0.83–1.06)	0.27	0.95 (0.85–1.05)	0.29	0.96 (0.86–1.06)	0.43
Gemini 2.5 Flash	Asian vs White	1.10 (0.95–1.27)	0.19	1.02 (0.89–1.18)	0.74	1.08 (0.95–1.23)	0.23
	Black vs White	1.02 (0.89–1.16)	0.78	0.94 (0.81–1.09)	0.41	0.96 (0.83–1.12)	0.62
	Hispanic vs White	1.19 (1.03–1.39)	0.02*	1.04 (0.90–1.20)	0.62	1.04 (0.90–1.19)	0.61
	Asian vs Hispanic	0.92 (0.80–1.06)	0.25	0.99 (0.85–1.15)	0.88	1.04 (0.90–1.20)	0.56
	Black vs Hispanic	0.85 (0.74–0.99)	0.03*	0.91 (0.79–1.04)	0.16	0.93 (0.81–1.07)	0.30
	Asian vs Black	1.08 (0.94–1.24)	0.27	1.09 (0.94–1.27)	0.27	1.12 (0.97–1.30)	0.11

Odds ratios and 95% confidence intervals (CIs) are estimated using generalized estimating equation models. Odds ratios compare the odds of a correct diagnosis between the first-listed and second-listed race/ethnicity groups. Odds ratios greater than 1.00 indicate higher diagnostic accuracy in the first-listed group, whereas odds ratios less than 1.00 indicate higher accuracy in the second-listed group. Numbers in parentheses are 95% CIs for the odds ratios

*p-values indicate statistical significance (*p* < 0.05)

For vision models, six significant race-related differences were observed among females under basic prompting. GPT-5 showed significantly lower accuracy for Black than White patients (OR 0.90; 95% CI 0.81–1.00; *p* = 0.0496) and Hispanic patients (OR 0.90; 95% CI 0.81–0.99; *p* = 0.04). Claude Sonnet 4.5 showed significantly lower accuracy for Asian than White patients (OR 0.91; 95% CI 0.83–1.00; *p* = 0.046) and Hispanic patients (OR 0.89; 95% CI 0.81–0.98; *p* = 0.02). Gemini 2.5 Flash showed significantly higher accuracy for Hispanic than White patients (OR 1.19; 95% CI 1.03–1.39; *p* = 0.02) and lower accuracy for Black than Hispanic patients (OR 0.85; 95% CI 0.74–0.99; *p* = 0.03). No significant race-related differences were observed among males under any prompting strategy or among females under self-consistency or chain-of-thought prompting (all *p* ≥ 0.08).

**Fig. 2:**
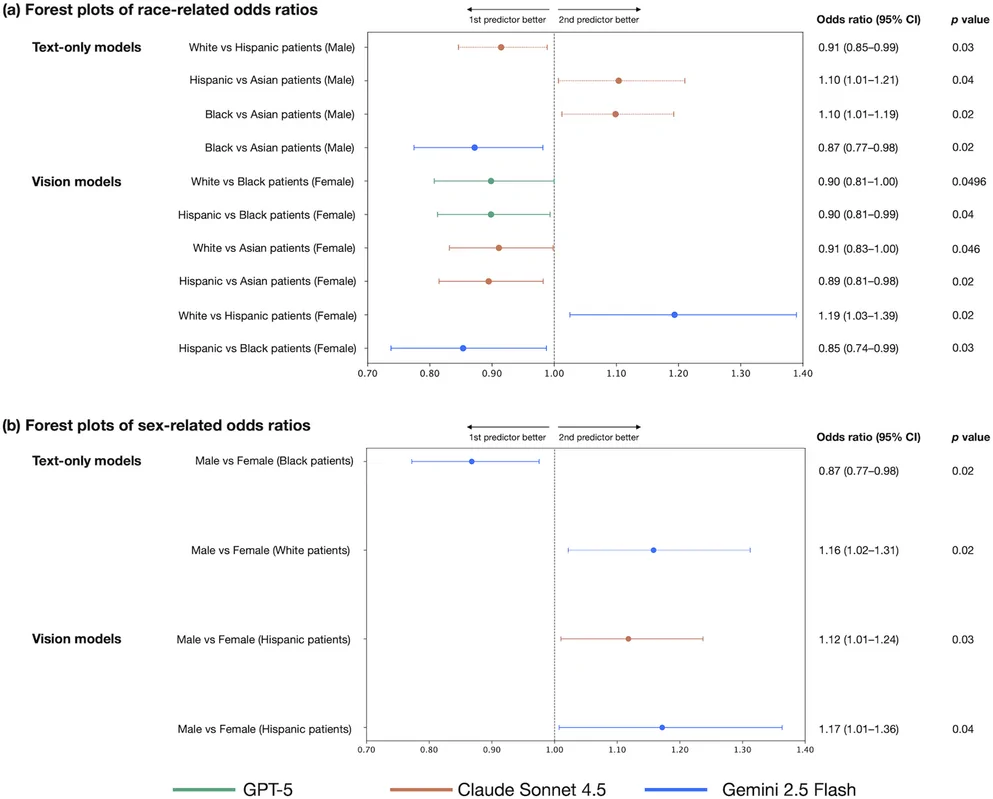
Forest plots show statistically significant race- and sex-related differences in diagnostic accuracy of three large language models (GPT-5, Claude Sonnet 4.5, and Gemini 2.5 Flash). Odds ratios (dots) and 95% confidence intervals (CIs) (horizontal lines) are displayed. Solid horizontal lines indicate basic prompting, and dotted horizontal lines indicate self-consistency prompting. **a** Forest plots of race-related odds ratios compare diagnostic accuracy between pairs of race/ethnicity groups. **b** Forest plots of sex-related odds ratios compare diagnostic accuracy between males and females within each race/ethnicity group. Odds ratios less than 1.00 indicate higher diagnostic accuracy in the first-listed group, whereas odds ratios greater than 1.00 indicate higher accuracy in the second-listed group

Overall, ten significant race-related differences were observed (Fig. 2). By race/ethnicity, the number of instances with significantly higher accuracy was as follows: Hispanic (4/10 [40%]), White (3/10 [30%]), Asian (2/10 [20%]), and Black (1/10 [10%]). The number of instances with significantly lower accuracy was as follows: Black (4/10 [40%]), Asian (3/10 [30%]), Hispanic (2/10 [20%]), and White (1/10 [10%]). Black patients were the most likely to have significantly lower accuracy and the least likely to have significantly higher accuracy among the four race/ethnicity groups. Across both text-only and vision models, vision models with female patients under basic prompting showed the highest frequency of significant race-related differences (6/10 [60%]; GPT-5 = 2, Claude Sonnet 4.5 = 2, Gemini 2.5 Flash = 2), compared with other prompting strategies, vision models with male patients, and all text-only models.

### Sex-related differences

For each of the text-only and vision models, 36 sex-based comparisons were analyzed. Table 4 shows sex-related differences in diagnostic accuracy by model, race/ethnicity, and prompting strategy (Fig. 2). For text-only models, Gemini 2.5 Flash showed significantly lower accuracy for females than males in Black patients under basic prompting (OR 0.87; 95% CI 0.77–0.98; *p* = 0.02) and significantly higher accuracy for females than males in White patients under self-consistency prompting (OR 1.16; 95% CI 1.02–1.31; *p* = 0.02). No other significant sex-related differences were observed (all *p* ≥ 0.06).

**Table 4:** Sex-related differences in diagnostic accuracy

Model	Race and ethnicity	Basic prompt		Self-consistency		Chain-of-thought	
		Odds ratio	*p* value	Odds ratio	*p* value	Odds ratio	*p* value
*Text-only model*							
GPT-5	Asian	0.94 (0.84–1.05)	0.27	1.06 (0.95–1.18)	0.33	0.89 (0.78–1.02)	0.08
	Black	0.97 (0.85–1.10)	0.64	0.92 (0.79–1.07)	0.26	1.04 (0.91–1.19)	0.58
	Hispanic	1.00 (0.88–1.13)	> 0.99	0.97 (0.87–1.08)	0.58	1.03 (0.91–1.17)	0.63
	White	1.00 (0.87–1.15)	> 0.99	0.98 (0.87–1.10)	0.70	0.95 (0.85–1.07)	0.41
Claude Sonnet 4.5	Asian	0.98 (0.91–1.05)	0.53	0.96 (0.88–1.06)	0.42	0.93 (0.86–1.00)	0.06
	Black	1.00 (0.92–1.08)	> 0.99	1.00 (0.93–1.07)	0.90	0.99 (0.92–1.07)	0.80
	Hispanic	1.02 (0.95–1.10)	0.61	1.06 (0.99–1.13)	0.08	0.96 (0.89–1.04)	0.28
	White	0.98 (0.91–1.05)	0.54	1.00 (0.91–1.08)	0.91	0.99 (0.91–1.07)	0.72
Gemini 2.5 Flash	Asian	1.10 (0.97–1.25)	0.14	1.07 (0.95–1.22)	0.27	0.95 (0.84–1.07)	0.41
	Black	0.87 (0.77–0.98)	0.02*	1.06 (0.94–1.21)	0.35	1.02 (0.91–1.15)	0.70
	Hispanic	0.97 (0.87–1.09)	0.62	1.02 (0.91–1.15)	0.75	0.97 (0.86–1.09)	0.58
	White	0.97 (0.87–1.08)	0.55	1.16 (1.02–1.31)	0.02*	0.89 (0.79–1.01)	0.08
*Vision model*							
GPT-5	Asian	0.95 (0.87–1.03)	0.21	1.04 (0.93–1.16)	0.50	0.99 (0.90–1.09)	0.85
	Black	1.00 (0.91–1.11)	0.93	0.98 (0.88–1.10)	0.74	1.01 (0.91–1.12)	0.86
	Hispanic	1.09 (0.99–1.20)	0.10	1.00 (0.91–1.11)	0.93	1.04 (0.94–1.14)	0.45
	White	1.05 (0.94–1.17)	0.36	0.96 (0.86–1.08)	0.51	0.97 (0.87–1.08)	0.60
Claude Sonnet 4.5	Asian	0.96 (0.87–1.05)	0.37	0.99 (0.89–1.11)	0.91	1.04 (0.96–1.13)	0.28
	Black	1.02 (0.91–1.14)	0.75	1.01 (0.91–1.12)	0.81	1.05 (0.93–1.18)	0.41
	Hispanic	1.12 (1.01–1.24)	0.03*	1.03 (0.94–1.12)	0.56	1.01 (0.93–1.10)	0.77
	White	1.03 (0.93–1.14)	0.56	1.01 (0.92–1.10)	0.89	0.99 (0.91–1.09)	0.90
Gemini 2.5 Flash	Asian	1.07 (0.96–1.20)	0.20	1.01 (0.88–1.16)	0.87	1.05 (0.91–1.21)	0.50
	Black	0.98 (0.84–1.13)	0.74	0.95 (0.83–1.09)	0.49	0.92 (0.80–1.07)	0.28
	Hispanic	1.17 (1.01–1.36)	0.04*	0.98 (0.86–1.11)	0.72	0.92 (0.80–1.05)	0.23
	White	0.96 (0.83–1.11)	0.56	1.00 (0.87–1.14)	> 0.99	0.94 (0.81–1.09)	0.41

Odds ratios and 95% confidence intervals (CIs) are estimated using generalized estimating equation models for female versus male, with male as the reference. Numbers in parentheses are 95% CIs

*p-values indicate statistical significance (*p* < 0.05)

For vision models, significant sex-related differences were observed in Hispanic patients under basic prompting, with higher accuracy for females than males in Claude Sonnet 4.5 (OR 1.12; 95% CI 1.01–1.24; *p* = 0.03) and Gemini 2.5 Flash (OR 1.17; 95% CI 1.01–1.36; *p* = 0.04). No other significant sex-related differences were observed (all *p* ≥ 0.10).

### Race- and sex-related differences by prompting strategy

**Fig. 3:**
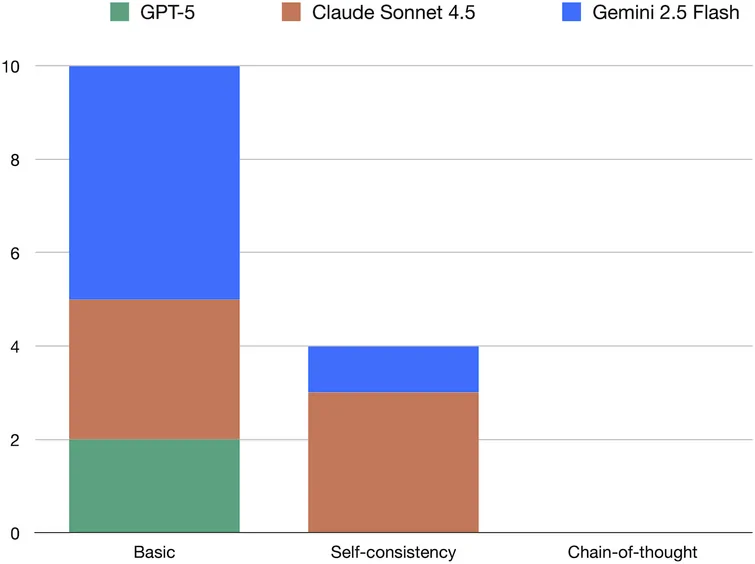
Bar graph shows the number of statistically significant race- and sex-related differences in diagnostic accuracy across three prompting strategies. With basic prompting, ten significant differences are observed across models (GPT-5: 2; Claude Sonnet 4.5: 3; Gemini 2.5 Flash: 5). With self-consistency prompting, four significant differences are observed (Claude Sonnet 4.5: 3; Gemini 2.5 Flash: 1). In contrast, with chain-of-thought prompting, no significant race- or sex-related differences are observed in any model

Figure 3 summarizes the number of statistically significant race- and sex-related differences in diagnostic accuracy under each prompting strategy. With basic prompting, ten significant differences were observed across models (GPT-5: 2; Claude Sonnet 4.5: 3; Gemini 2.5 Flash: 5). With self-consistency prompting, four significant differences were observed (Claude Sonnet 4.5: 3; Gemini 2.5 Flash: 1). In contrast, with chain-of-thought prompting, no significant race- or sex-related differences were observed in any model.

## Discussion

This study addresses the lack of systematic evaluation of demographic bias in LLMs increasingly integrated into diagnostic radiology. Analyzing 123,552 responses from three LLMs (GPT-5, Claude Sonnet 4.5, and Gemini 2.5 Flash) across 286 *Diagnosis Please* cases each given eight race-sex scenarios, we identified both race/ethnicity-related and sex-related differences in diagnostic accuracy. Black patients were disproportionately affected, accounting for 40% (4/10) of instances with significantly lower accuracy among the four race/ethnicity groups. Additionally, vision models with basic prompting for female patients exhibited the largest number of significant race-related differences (60% [6/10]). While diagnostic accuracy did not differ significantly across prompting strategies for either text-only or vision models, prompting strategy influenced bias mitigation: basic and self-consistency prompting yielded ten and four significant demographic-related differences, whereas chain-of-thought prompting yielded no significant differences. To our knowledge, this is the first study to evaluate racial and sex biases in LLMs for diagnostic radiology and to investigate the effectiveness of prompting strategies for mitigating these biases. Identifying and addressing these biases is essential, as unrecognized algorithmic bias may lead to inequitable care and exacerbate health disparities [9, 10].

This study revealed that LLMs can exhibit racial and sex biases in diagnostic radiology and suggested a diagnostic bias that may disproportionately disadvantage Black patients compared with other race/ethnicity groups. A previous study has reported that GPT-4o perpetuates race- and sex-based stereotypes and skews differential diagnoses across a range of clinical conditions [11]. Other studies have shown that LLM-generated medical recommendations vary by race, sex, and other sociodemographic factors, raising concerns that Black patients and other socially marginalized groups may experience systemic, medically unjustified inequities and disadvantages [13, 15, 26, 27]. In radiology, LLMs have been reported to exhibit racial bias when simplifying radiology reports, with summaries for Black patients written at a significantly lower reading grade level [28]. Our findings align with previous studies and indicate that such biases can also arise in diagnostic radiology.

This study evaluated demographic bias using a counterfactual design in which only demographic information was varied while all other clinical content was held constant. Given this design, although the significant odds ratios for race- and sex-related differences were modest in magnitude (ranging from 0.85 to 1.19), these effect sizes suggested a systematic influence of demographic information alone on model performance, which could produce meaningful disparities at the population level. To situate these findings in relation to overall model capability, the diagnostic accuracy of the evaluated LLMs should be considered alongside previously reported benchmarks of LLM and radiologist diagnostic performance. In previous studies using *Diagnosis Please* cases, GPT-4 achieved diagnostic accuracies of 54% and 15% in the text-only and vision models, respectively, and radiologists achieved an accuracy of 48% based on medical history and image interpretation [5, 21]. The models evaluated in this study were more recent than those evaluated in previous studies and showed diagnostic accuracies comparable to or higher than those previously reported for both the text-only and vision models; therefore, their diagnostic performance appears reasonable as a basis for interpreting the demographic bias observed in this study. It should be noted that among the vision models, Claude Sonnet 4.5 and Gemini 2.5 Flash showed absolute diagnostic accuracies which were lower than the previously reported diagnostic accuracy of radiologists. Given these low diagnostic accuracies, some of the observed race- and sex-related differences may partly reflect artifacts of low baseline accuracy rather than systematic bias.

Demographic bias manifested differently between text-only and vision models, with racial bias more pronounced in vision models for female patients. Previous studies have reported that vision-language models are insufficiently reliable for direct image interpretation and that their performance depends more on the medical history and descriptions of imaging findings than on the images themselves [4, 6, 29]. When the medical history and images are provided to vision models, their diagnostic reasoning tends to rely more on the medical history; therefore, vision models may be more likely to exhibit demographic bias driven by demographic information in the medical history than text-only models. Moreover, vision models showed significant race-related differences for females but not males. One potential explanation is that, compared with males, females may have been disproportionately represented across race/ethnicity in the LLM training data, leading to racial bias that manifests more frequently in female patients [7, 8, 13, 27]. Another potential explanation is that placing demographic information at the beginning of each medical history could have increased the influence of demographic descriptors and amplified the effects of imbalances in the training data. In addition, demographic information may have influenced how vision models integrated medical history with image-derived information. Thus, the more pronounced racial bias in vision models for female patients may reflect multiple interacting factors rather than imbalances in the training data alone, and further studies are needed to clarify these mechanisms.

This study also demonstrated that demographic biases in LLMs for diagnostic radiology varied across prompting strategies and suggested that chain-of-thought prompting may mitigate these biases. Although diagnostic accuracy did not differ significantly across prompting strategies, the frequency of significant race- and sex-related differences varied by prompting strategy, indicating that chain-of-thought prompting may represent a useful strategy for mitigating demographic bias without affecting diagnostic accuracy. Various bias mitigation strategies for LLMs have been explored [30, 31]. Prompt engineering has been shown to substantially influence model outputs [3, 4, 16], and implementing appropriate prompting strategies may represent a simple yet effective approach to mitigating biases [13]. Chain-of-thought prompting requires LLMs to articulate stepwise reasoning, enabling more comprehensive and well-reasoned responses [24, 32]; therefore, this strategy may have helped mitigate demographic bias. Additionally, chain-of-thought prompting makes model reasoning explicit, allowing clinicians to evaluate the model’s diagnostic process and thereby enabling human oversight to further mitigate bias.

This study had several limitations. First, we evaluated the diagnostic performance of LLMs using *Diagnosis Please* cases, which may not fully reflect real-world radiology practice. Furthermore, because *Diagnosis Please* cases were publicly available online before the knowledge cutoffs of the evaluated models, these cases were likely included in the training data of these LLMs, potentially inflating model performance and affecting bias evaluation. Thus, a sensitivity analysis of demographic bias restricted to cases not included in the LLMs’ training data was not feasible in this study. Such training data contamination may have inflated baseline diagnostic accuracy, likely affecting model performance more than the demographic comparisons themselves, because the counterfactual design modified only race/ethnicity and sex while holding the clinical content constant. Second, this study was designed to evaluate demographic bias by comparing diagnostic accuracy across race-sex scenarios in which race/ethnicity and sex information was varied. However, some counterfactual scenarios may have been clinically less plausible. Additionally, some of the observed demographic-related differences may reflect epidemiologically grounded reasoning rather than demographic bias, and the present study design could not fully distinguish between these possibilities. Therefore, interpreting the observed demographic-related differences as definitive evidence of demographic bias may be premature. Third, we did not evaluate the condition in which race/ethnicity and sex information was removed. Such a condition could provide an additional reference for assessing how explicit demographic information affects model performance. Future studies should incorporate such demographic-masked conditions to further clarify the influence of demographic attributes on model performance. Fourth, we focused on basic demographic attributes. Future research should include additional race/ethnicity groups, gender identity, and other sociodemographic factors to more comprehensively assess diagnostic bias in radiology. Fifth, we fixed the LLM temperature at 1.0 and did not evaluate other settings. Higher temperatures increase output diversity and creativity, whereas lower temperatures increase determinism and reproducibility. However, a previous study reported little difference in race/sex-based diagnostic biases across the temperature range of 0–1.0, suggesting minimal influence of this parameter on our findings [11]. Finally, because we performed multiple statistical comparisons without adjustment for multiplicity, this study might overestimate the demographic biases in LLMs. However, as this was an exploratory study aimed at comprehensively identifying potential demographic biases, we prioritized maintaining sensitivity in detecting demographic-related differences.

In conclusion, this study demonstrated that LLMs exhibited racial and sex biases in diagnostic radiology and suggested that chain-of-thought prompting may help mitigate these demographic biases. While LLMs have considerable potential to assist diagnostic radiology, this study highlighted the need for ongoing evaluation and mitigation of biases in LLMs to avoid introducing or perpetuating health inequities in clinical practice.
